# Phylomitogenomics reconfirm the phylogenetic position of the genus *Metaplax* inferred from the two grapsid crabs (Decapoda: Brachyura: Grapsoidea)

**DOI:** 10.1371/journal.pone.0210763

**Published:** 2019-01-25

**Authors:** Jianqin Chen, Yuhui Xing, Wenjia Yao, Xue Xu, Chenling Zhang, Zhenhua Zhang, Qing Liu

**Affiliations:** 1 Institute of Aquatic Biology, Jiangsu Key Laboratory of Biofunctional Molecule, School of Life Sciences, Chemistry & Chemical Engineering, Jiangsu Second Normal University, Nanjing, China; 2 Nanjing Normal University Zhongbei College, Nanjing, China; 3 College of Life Sciences, Nanjing Normal University, Nanjing, China; The Chinese University of Hong Kong, CHINA

## Abstract

Two new complete mitogenomes of the grapsids, *Metaplax longipes* Stimpson, 1858 and *Nanosesarma minutum* (De Man, 1887) were sequenced using next-generation sequencing (NGS). By analyzing a combination of 75 Brachyura taxa, our phylomitogenomic inferences suggested that *Metaplax* crab seperated earlier from the sesarmid crabs and closely related to the varunids with respect to *Nanosesarma* crab. It reconfirmed that the *Metaplax* should be removed from the Sesarmidae and assinged to the Varunidae. Additional mitogenomic comparisons including gene rearrangement and genomic organization were conducted among the 33 taxa of Grapsoidea and Ocypodoidea, and a shared rearrangement pattern between *Metaplax longipes* and the varunids were recovered, which also strongly supported the inference for the phylogenetic position of the *Metaplax*.

## Introduction

The genus *Metaplax* H. Milne Edwards, 1852, which currently contains 12 species [1], along with three other genera, *Cyclograpsus*, *Chasmagnathus* and *Helice*, found in the China Seas, were historically assigned to the Sesarminae [2–3], based on the presence of a hairy crest on the third maxilliped and distinct characters in the pterygostomian and ventrolateral regions of the carapace [3–6]. However, the cladistic analysis proposed by Sternberg and Cumberlidge [7] found that *Metaplax* and the five genera *Chasmagnathus* De Haan, 1833, *Helice* De Haan, 1833; *Cyclograpsus* H. Milne Edwards, 1837; *Paragrapsus* H. Milne Edwards, 1853; and *Helograpsus* Campbell & Griffin, 1966 fail to conform to the other sesarmine genera. The early molecular phylogenetic analysis based on partial sequences of 12S rRNA and/or 16S rRNA revealed that these six genera were more closely related to other varunid crabs and suggested that *Metaplax* and the five related genera were excluded from the Sesarminae and were included in the Varuninae [8–11]. The validity of the character of a hairy crest on the 3^rd^ maxilliped for intergeneric taxonomy, therefore, needs to be reevaluated, as proposed by Schubart et al [8, 9]. The morphological characters, such as the adult morphological characters, i.e., the location of the male genital openings [12], typically a stridulatory suborbital crest that is relatively straight and extends some distance across the lateral branchial region [13] and several larval characters, i.e., the 2, 2 seta l pattern on the endopod of the maxilla and the type of antenna and telson [14], provide strong support that these genera are included to the Varunidae. Therefore, these six genera, including *Metaplax*, combined with *Helicana* K. Sakai & Yatsuzuka, 1980, in the updated system of the Brachyurans of the world, were assigned to the Cyclograpsinae and Grapsidae by Davie in 2002 [13] and, more recently, to the Cyclograpsinae, Varunidae, and Grapsoidea by Ng et al. in 2008 [1]. However, the updated position of the genus *Metaplax* that proposed by Ng et al. was not commonly accepted, as in recent years the genus still has been included within the Grapsidae rather than in the Varunidae in some researchs [15, 16]. The phylogenetic position of the *Metaplax* needs to be further confirmed.

Mitogenomic data, including both primary sequences and gene rearrangements, have been shown to contain useful phylogenetic information for inferring the evolutionary relationships among eubrachyurans [17–22]. Compared to the data on the partial fragments of genes, phylogenetic reconstruction using mitogenomic data could help avoid the interference of nuclear pseudogenes [23]. Gene order patterns have the potential to act as synapomorphies for specific lineages and taxonomic groups, providing support for the hypotheses for phylogenetic relationships [21, 24]. Here, to reconfirm the taxonomic assignment of the genus *Metaplax*, we first sequenced and annotated the complete mitogenome of *Metaplax longipes* Stimpson, 1858. We also added the mitogenome of *Nanosesarma minutum* (De Man, 1887), representing the first species from the *Nanosesarma* (Grapsoidea: Sesarmidae), not only to increase the taxon, but also to make additional comparisons in the nucleotide contents and the gene rearrangements of mitogenome among related groups by which to provide new evidence for taxonomic position of the *Metaplax*. As typical semiterrestial species, Grapsoidea live in mangrove swamps or on rocky shores, muddy and sand shores or other swampy grouds, but in this kind of large ecological regions there are various niches [3]. For example, the *M*. *longipes* likes intertidal mud flats and sand beach, while the *N*. *minutum* prefers to living on the muddy ground or under stones. Obtaining a stable phylogenetic position of *M*. *longipes* and *N*. *minutum* based on mitogenomic data will help us to understand the their habitatal preference.

## Materials and methods

### Ethics statement

No specific permits were required for crab collection and research in the selected locations. The sampling locations were not privately owned or protected in any way. The crabs used for the experiments were not considered protected or endangered, and their collection is legal in China.

### Specimen collection and identification

*M*. *longipes* and *N*. *minutum* were collected by hand in mangrove of Golden gulf, Beihai (21°25'27.74"N, 109°13'3.20"E), Guangxi Province and in Mawei Seafood Market, Fuzhou (25°59'45.76"N, 119°28'40.63"E), Fujian Province, China, respectively. Both specimens were preserved in 95% ethanol and deposited at the Jiangsu Key Laboratory of Biofunctional Molecule (Jiangsu Second Normal University), School of Life Sciences, Chemistry & Chemical Engineering. Identification was performed morphologically with a stereo dissecting microscope according to the morphological information on crabs of the China Seas [3].

### DNA extraction and sequencing

Total genomic DNA was extracted using a Cell and Tissue DNA Extraction Kit according to the manufacturer’s instructions (Generay Biotech, Shanghai, China). The quality of the extracted DNA was examined by 1% agarose gel electrophoresis and was then sent to Novogene (Beijing, China) for sequencing using the Illumina MiSeq Desktop Sequencer (2 × 150 bp paired-end reads). The average insert size of the sequencing libraries was approximately 300 bp.

### Mitochondrial genome assembly and annotation

Mitochondrial genome assemblies were conducted in Geneious 11.1.2 using parameter settings described previously [22, 25]. Protein-coding genes (PCGs) were identified by finding the ORFs (https://www.ncbi.nlm.nih.gov/orffinder) using the invertebrate mitochondrial genetic code. The secondary structure and anticodons of transfer RNA (tRNA) genes were identified using the results of both tRNAscan-SE [26] and MITOS Web Server (http://mitos2.bioinf.uni-leipzig.de/index.py) analyses [27]. Similarly, the large and small rRNA subunits (*srRNA* and *lrRNA*) were determined using MITOS Web Server or alignment to closely related references. The graphical map of the mitogenomes was drawn using the online mitochondrial visualization tool Organellar Genome DRAW (https://chlorobox.mpimp-golm.mpg.de/OGDraw.html) [28]. The nucleotide composition was calculated in MEGA 6.0 [29]. The mitogenomes of *M*. *longipes* and *N*. *minutum* were deposited under the following respective GenBank accession numbers: MH899176 and MH899177.

### Phylogenetic analysis

To infer the phylogenetic position of the studied species, we used a 75-taxon set as ingroups from divergent lineages comprising the 55 genera, 25 families and 15 superfamilies of Brachyura (S1 Table). Eight species from the Gebiidea and Anomura were also included in the analyses as outgroups (S1 Table). After removing all termination codons, the putative amino acid (AA) sequences of the 13 PCGs and sequences for two rRNAs were individually aligned using MAFFT 7.215 [30]. The ambiguously aligned regions from each gene were removed by Gblocks V. 0.91b with default settings [31]. The pruned, aligned AA sequences were then used as a backbone to align the corresponding nucleotide (NT) sequences using DAMBE 5.3.15 [32]. The final NT data was composed of a concatenation of the 13 PCGs and two rRNAs. We then conducted phylogenetic analyses using Maximum likelihood (ML) and Bayesian Inference (BI). The best-fit partitioning schemes and the substitution model for each partition were selected by PartitionFinder 1.1.1 [33] and ModelFinder [34] using a greedy search with Bayesian information criteria (BIC). The ML tree was reconstructed in IQTREE v1.6.3 with 1,000 ultrafast bootstrap (BS) replicates [35, 36]. Bayesian inference (BI) was performed using MrBayes 3.2.2 [37] through the Cipres Science Gateway [38]. The Markov chain Monte Carlo (MCMC) was started with one million generations, with every 1000 generations sampled, and the first 25% of the generations were discarded as burn-in. The standard deviation of split frequencies was 0.0089 (below 0.01) after 1,500,000 generations, which reflected that the two runs strongly indicated convergence.

## Results

### Mitogenomic general characters

**Fig 1:**
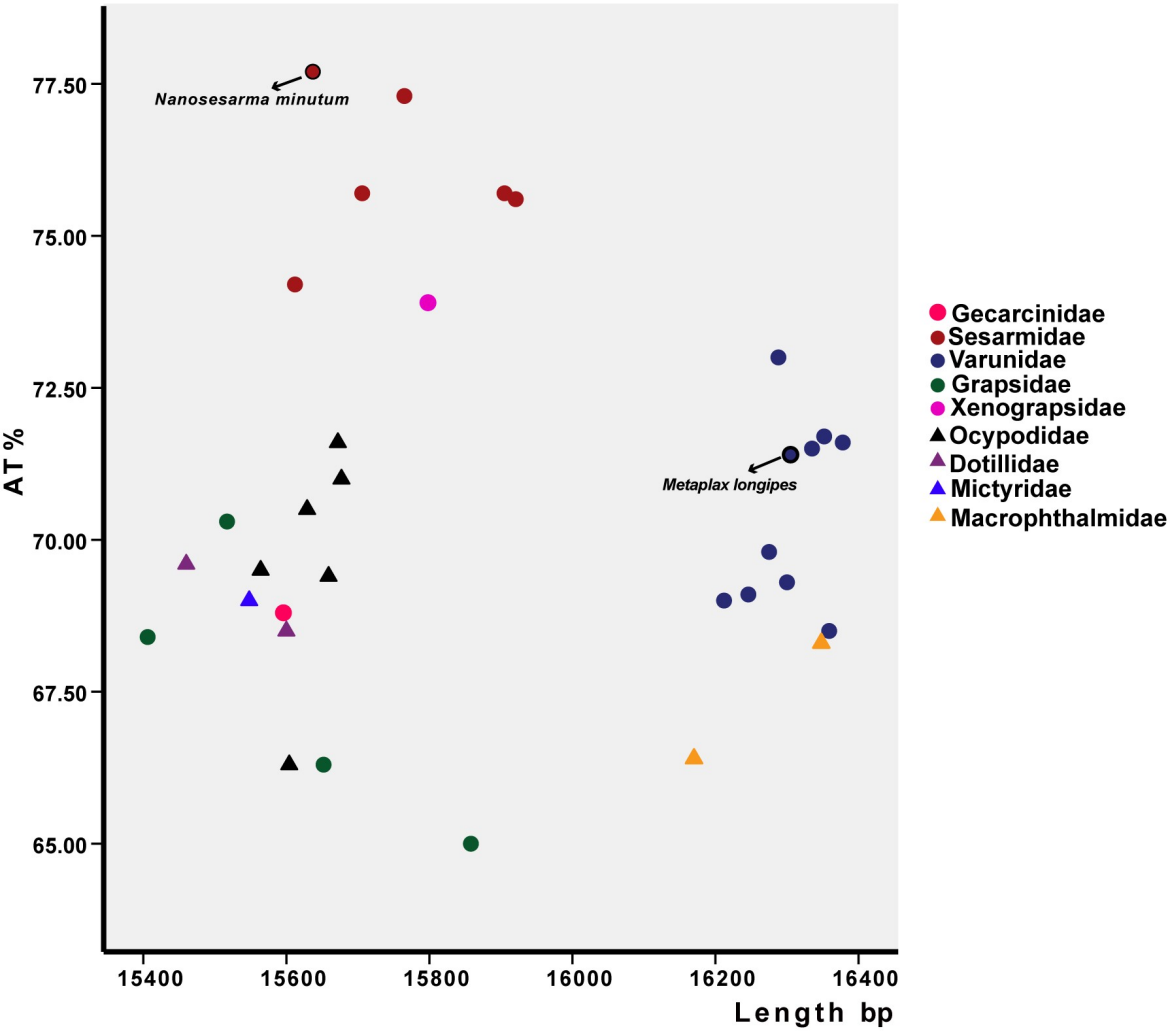
Length and A+T content of the mitogenomes of 33 taxa from the Grapsoidea and Ocypodoidea. The nine families studied are represented by different colors. Circles represent Grapsoidea, while triangles depict Ocypodoidea.

The mitogenomes of *M*. *longipes* and *N*. *minutum* were determined to contain an entire set of 37 genes plus a larger main noncoding region (mNCR), and were 16,305 and 15,637 bp in length, respectively (Table 1; Fig 1). Notably, the length of the *M*. *longipes* mitogenome was similar to that of the varunid crabs sampled (ranging from 16,170 to 16,212 bp; Fig 1; S2 Table). We found that the length of varunid crabs mitogenomes was longer than the average length of the other grapsid mitogenomes (15,698 bp) and ranged from 15,406 to 15,920 bp. The length of the *N*. *minutum* mitogenome located within the characteristic size of most of the sesarmids (15,612 to 15,920 bp). The A+T content of the *M*. *longipes* mitogenome was 71.40%, which is similar to the average value for varunid crabs (avg. 70.39%, ranging from 68.5 to 73.0%; Fig 1; S2 Table), while the A+T content of the *N*. *minutum* mitogenome was the highest among the sesarmid crabs (avg. 76.03%), which is greater than the average value for the other grapsids, even ocypodid crabs.

**Table 1:** Mitogenomic features of *Nanosesarma minutum* and *Metaplax longipes*.

Feature	*Nanosesarma minutum*			Feature	*Metaplax longipes*		
	Position	Length(bp)	IGN*		Position	Length(bp)	IGN*
*cox1*	1–1539	1539	-5	*cox1*	1–1539	1539	-5
*trnL2*	1535–1603	69	7	*trnL2*	1535–1600	66	8
*cox2*	1611–2298	688	0	*cox2*	1609–2313	705	55
*trnK*	2299–2367	69	0	*atp8*	2369–2530	162	-7
*trnD*	2368–2435	68	0	*atp6*	2524–3198	675	-1
*atp8*	2436–2594	159	-4	*cox3*	3198–3989	792	-1
*atp6*	2591–3262	672	7	*trnG*	3989–4052	64	0
*cox3*	3270–4061	792	-1	*nad3*	4053–4403	351	5
*trnG*	4061–4124	64	-3	*trnA*	4409–4473	65	8
*nad3*	4122–4475	354	2	*trnR*	4482–4543	62	1
*trnA*	4478–4541	64	7	*trnN*	4545–4609	65	0
*trnR*	4549–4612	64	0	*trnS1*	4610–4702	93	0
*trnN*	4613–4678	66	1	*trnT*	4703–4768	66	0
*trnS1*	4680–4747	68	1	*trnP*^#^	4769–4834	66	11
*trnE*	4749–4815	67	1	*nad1*^#^	4846–5778	933	40
*trnH*^#^	4817–4880	64	1	*trnL1*^#^	5819–5885	67	0
*trnF*^#^	4882–4947	66	4	*lrRNA*^#^	5886–7238	1353	70
*nad5*^#^	4952–6664	1713	50	*srRNA*^#^	7309–8208	900	0
*nad4*^#^	6715–8064	1350	-7	*trnH*^#^	8209–8272	64	41
*nad4L*^#^	8058–8360	303	8	*nad5*^#^	8314–10044	1731	71
*trnT*	8369–8434	66	0	*trnV*^#^	10116–10186	71	0
*trnP*^#^	8435–8499	65	2	*mNCR*	10187–11096	910	0
*nad6*	8502–9005	504	-1	*trnQ*	11097–11165	69	13
*cob*	9005–10139	1135	0	*trnC*^#^	11179–11243	65	8
*trnS2*	10140–10205	66	17	*trnY*^#^	11252–11320	69	8
*nad1*	10223–11161	939	33	*trnK*	11329–11399	71	-2
*trnL1*^#^	11195–11261	67	0	*trnD*	11398–11467	70	9
*lrRNA*^#^	11262–12580	1319	0	*trnE*	11477–11544	68	5
*trnV*^#^	12581–12653	73	0	*trnF*	11550–11613	64	13
*srRNA*^#^	12654–13489	836	0	*nad4*^#^	11627–12970	1344	-7
*mNCR*	13490–14186	697	0	*nad4L*^#^	12964–13266	303	64
*trnQ*^#^	14187–14255	69	14	*nad6*	13331–13849	519	-1
*trnI*	14270–14336	67	18	*cob*	13849–14983	1135	0
*trnM*	14355–14424	70	0	*trnS2*	14984–15409	66	38
*nad2*	14425–15432	1008	0	*trnI*	15088–15152	65	0
*trnW*	15433–15501	69	4	*trnM*	15153–15224	72	0
*trnC*^#^	15506–15572	67	0	*nad2*	15225–16235	1011	-2
*trnY*^#^	15573–15637	65	0	*trnW*	16234–16302	69	3
overall	15637	15481	156	overall	16305	15860	445

*IGN: intergenic nucleotide; negative numbers indicate that adjacent genes overlap.

^#^ Indicates the gene is encoded on the opposite strand.

### Gene rearrangement

**Fig 2:**
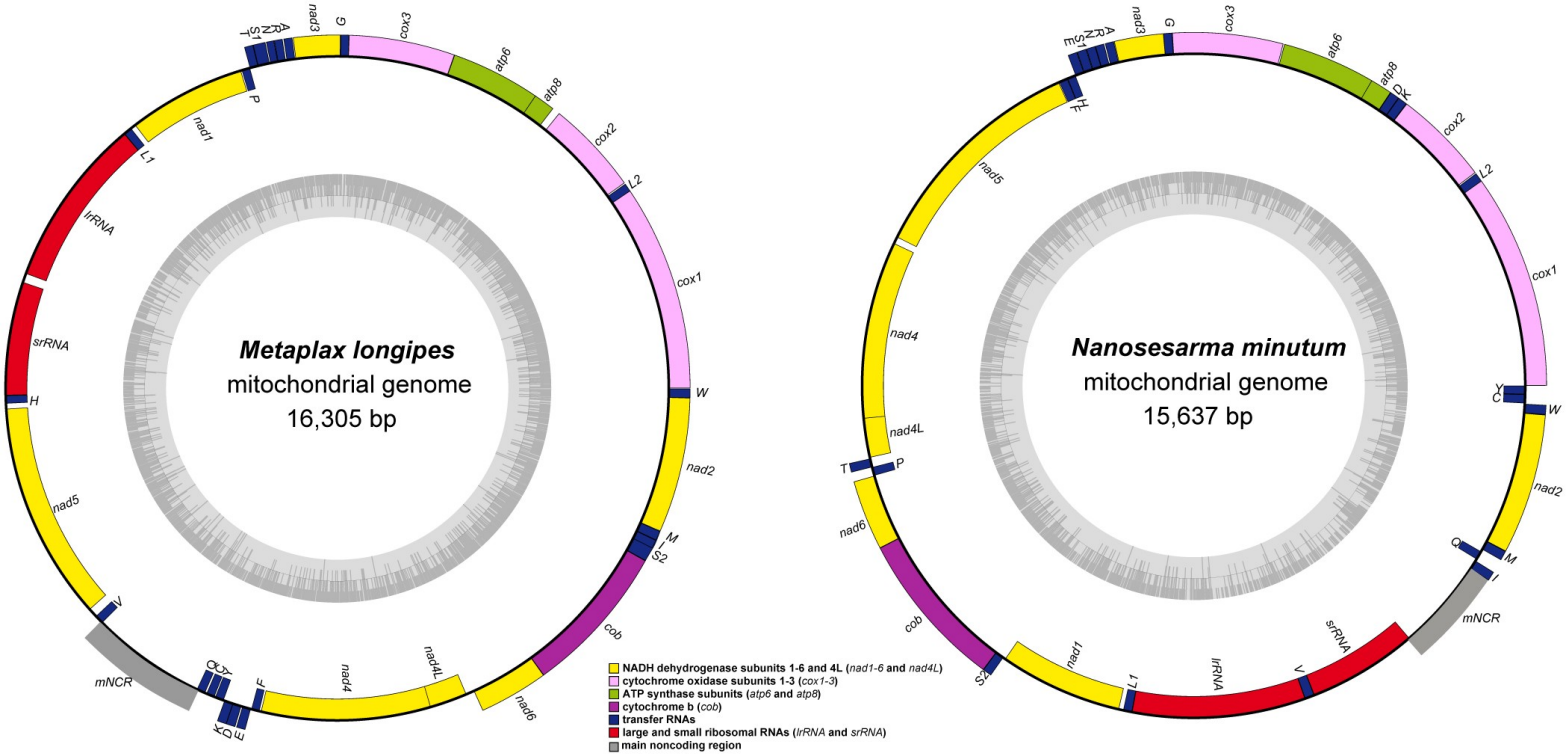
Mitogenomic maps of *Metaplax longipes* and *Nanosesarma minutum*. Genes are color-coded, i.e., cytochrome oxidase subunits 1–3 (*cox1*-*3*): pink, NADH dehydrogenase subunits 1–6 and 4L (*nad1*-*6* and *nad4L*): yellow, ATP synthase subunits (*atp6* and *atp8*): green, cytochrome b (*cob*): purple, large and small ribosomal RNAs (*lrRNA* and *srRNA*): red, transfer RNA genes (tRNA): blue, and the main noncoding region (mNCR): gray. Each tRNA gene is designated by a single-letter amino acid code except for *L1* (trnLeu (CUN)), *L2* (trnLeu (UUR)), *S1* (trnSer (AGN)), and *S2* (trnSer (UCN)).

Of the two species presented in this study, *M*. *longipes* presented a major gene rearrangement pattern identical to that of varunid crabs, including three tRNA gene pairs (*C*-*Y*, *K-D*, and *E-**F*), two tRNA genes (*Q* and *V*) and a *mNCR* translocated into the *nad5*-*nad4* gene junction; furthermore, a four-gene block (*nad1*-*L1*-*lrRNA*-*srRNA*) and a tRNA gene pair (*T*-*P*) were rearranged into the location between *nad3* and *nad5*, while *N*. *minutum* exhibited a typical tRNA gene cluster (*Q*-*I*-*M*) rearrangement identical to that of the other five sesarmid crabs (Fig 2).

### Phylogenetic analysis

**Fig 3:**
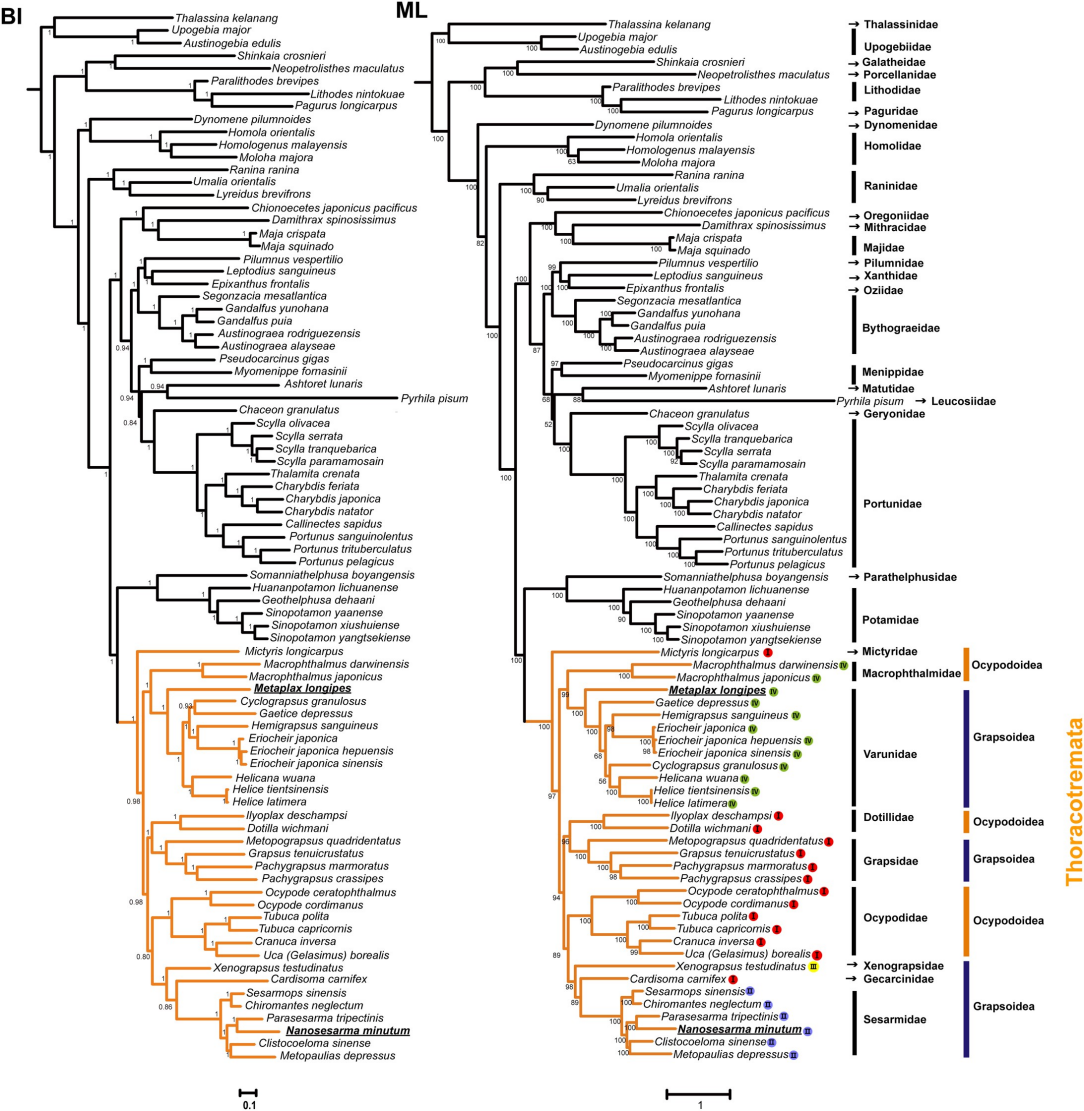
Trees show phylogeny of Brachyura using Bayesian inference (BI) and Maximum likelihood method (ML), based on the 13 protein-coding genes and two rRNAs. Values at the branches represent Bayesian posterior probabilities (BPP)/ ultrafast bootstrap values (BS). Within Thoracotremata, the codes (I–IV) behind tips of the branches correspond to the gene rearrangement patterns listed in Fig 4, and the mitogenomes sequenced in this study are underlined and bold.

The ML and BI phylogenetic trees produced similar topologies, but slightly different in several species (i.e. *Dynomene pilumnoides*, *Scylla serrate*, *Cyclograpsus granulosus*, and *Gaetice depressus*) (Fig 3). Within Thoracotremata, the samples from Grapsoidea and Ocypodoidea did not form monophyletic groups. For example, *Macrophthalmus* species as a distinct lineage from other ocypodids that was sister to grapsid species with high support (BPP = 1.00, BS = 99). As well, the two dotillid crabs were more closely related to Grapsoidea than Ocypodoidea, with strong nodal support in the BI analysis (BPP = 1.00) and ML analysis (BS = 96). As to the grapsid species newly presented in these trees, the *M*. *longipes* separated from the sesarmid crabs and formed a clade with other eight publicly available species of Varunidae, while *N*. *minutum* is sister to *Parasesarma tripectinis* (Shen, 1940) and then formed a strongly supported monophyletic clade (BPP = 1.00, BS = 100) with other four species from Sesarmidae.

## Discussion

**Fig 4:**
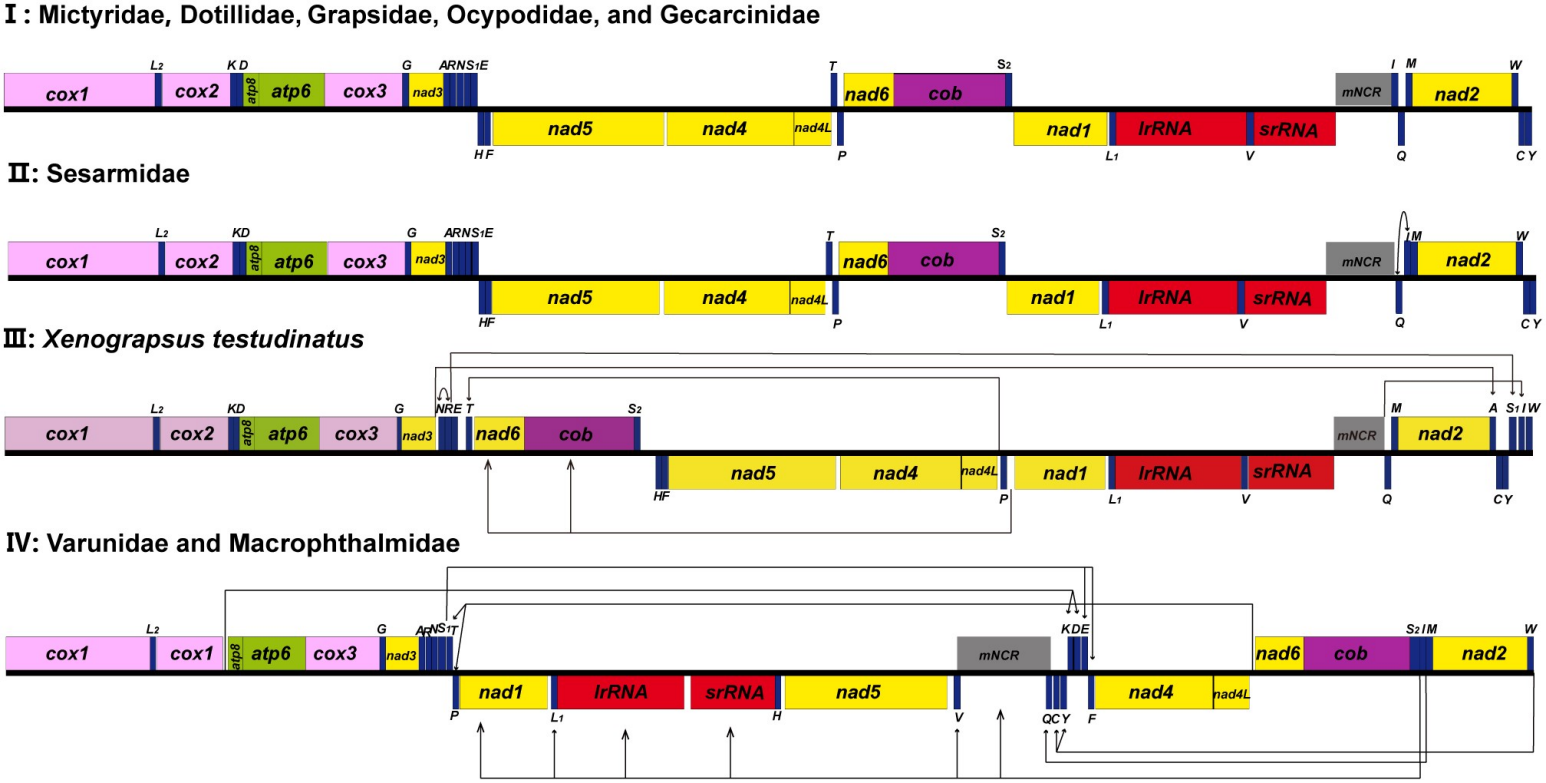
Gene rearrangements within the Grapsoidea and Ocypodoidea. Linear representation of the four patterns of gene rearrangements (I–IV). The transposition routes of the gene rearrangements are marked by lines and arrows.

Comparisons of mitogenomes, usually using both gene orders and DNA sequences (PCGs and the two rRNAs), provide strong support for brachyuran phylogenies [17, 18, 21–23, 39]. The length of the mitogenome is a basic character for each animal organism, with the entire set of 37 genes (except for several groups), which are closely packed and probably reflect a strong purifying selection over the evolutionary history, being highly conserved [17, 40, 41]. However, it was found here that there was an increase in the genome size of the mitogenome of the *Metaplax* crab. In addition, this length is similar to that found in the varunid crabs known to date. Further comparisons of gene orders and intergenic spacers revealed a consistent gene rearrangement pattern shared by the *Metaplax* and varunid crabs (Figs 3 and 4) with concordant multiple minor noncoding regions scattered in the mitogenomes. The genome organization with multiple intergenic spacers was first discovered in a varunid crab, *Eriocheir*, and was recognized as retention resulting from gene duplication and incomplete deletion, which further resulted in the gene rearrangement and increased genome size [17].

A recent reevaluation of the partial mtDNA sequences and morphological data for the phylogenetic position of *Metaplax* and other related crabs suggested that contrary to earlier studies positing this genus in the Sesarmidae family of grapsids, this genus presented a closer relationship with varunid genera [8–12, 14, 42]. We report here the first complete mitogenome of *Metaplax* crabs. Our mitogenome-based phylogenetics indicate that *M*. *longipes* was closely related to the other representative varunid crabs and was separated earlier from the “Sesarmidae” and “Grapsidae” clades. This result strongly supported the suggestion that *Metaplax* should be removed from Sesarmidae [8–11], and assigned to Varunidae [1]. This relationship was also supported by the shared gene rearrangements among *Metaplax* and the varunid species sampled and genome organization recovered in the present study. The result has helped to clarify that the morphologies located at the 3^rd^ maxilliped are not a synapomorphy for supporting the genus *Metaplax* as a taxon within the Sesarmidae, which appear to have symplesiomorphy.

Previous studies have pointed out there are four distinct patterns of gene rearrangements in the available mitogenomes from Grapsoidea and Ocypodoidea (Fig 4) [39]. Gene order patterns can act as synapomorphies for specific lineages at family level, and show potential in providing additional phylogenetic markers [21, 24]. In this study, the 2^nd^ pattern of gene rearrangement shared by all available mitogenomes from Sesarmidae. Noticeably, species from different superfamilies (Ocypodoidea and Grapsoidea) shared the 4^th^ gene rearrangement pattern, i.e. Macrophthalmidae and Varunidae, which supports a sister relationship presented in phylogenetic trees. The result also agreed with the previous suggestion that the polyphyly of Grapsoidea and Ocypodoidea [39, 43]. Considering that only species from Grapsoidea and Ocypodoidea within Thoracotremata were sampled, the validity of their relationship needs to be reconfirmed by further taxonomic sampling.

## Conclusion

In this study, we first determined the complete mitogenomes of the grapsid crabs *M*. *longipes* and *N*. *minutum*. Further, phylomitogenomic inferences suggested that *N*. *minutum* formed a clade with other sesarmids, while the *M*. *longipes* seperated earlier from the sesarmid clade. It reconfirmed that the *Metaplax* should be removed from the Sesarmidae and assinged to the Varunidae that proposed by Ng et al. or even older literature [1, 8–11]. New evidence that a consistent rearrangement pattern between *M*. *longipes* and the varunids was recovered, which also strongly supported the inference for the phylogenetic position of the *Metaplax*.

## Supporting information

S1 Table List of species used for phylogenetic analysis. (DOCX)

S2 Table The lengths and A+T contents of the mitogenomes of 33 taxa from the Grapsoidea and Ocypodoidea. (DOCX)
