# Supplementary material for: Phylomitogenomics reconfirm the phylogenetic position of the genus Metaplax inferred from the two grapsid crabs (Decapoda: Brachyura: Grapsoidea)
Source: PLoS One. 2019 Jan 25;14(1):e0210763. doi: 10.1371/journal.pone.0210763 (PMC6347246; doi:10.1371/journal.pone.0210763)
Supplement: S1 Table — (DOCX) [file pone.0210763.s001.docx]

**Table S1. List of species used for phylogenetic analysis.**

| **Intgroups** | | **Superfamily** | **Family** | **Genus** | **Species** | **Length (bp)** | **Accession No.** | **Reference** |
| --- | --- | --- | --- | --- | --- | --- | --- | --- |
| **Podotremata** | | Dromiidea | Dynomenidae | *Dynomene* | *Dynomene pilumnoides* | 16475 | KT182070 | [44] |
|  |  | Homolidea | Homolidae | *Homologenus* | *Homologenus malayensis* | 15793 | NC_026080 | [45] |
|  |  |  | Homolidae | *Moloha* | *Moloha majora* | 15903 | NC_029361 | [44] |
|  |  |  | Homolidae | *Homola* | *Homola orientalis* | 16084 | KT182071 | [44] |
|  |  | Raninoidea | Raninidae | *Umalia* | *Umalia orientalis* | 15466 | KM365084 | [46] |
|  |  |  | Raninidae | *Lyreidus* | *Lyreidus brevifrons* | 16112 | NC_026721 | [46] |
|  |  |  | Raninidae | *Ranina* | *Ranina ranina* | 15557 | NC_023474 | [47] |
| **Eubrachyura** | **Thoracotremata**  **Heterotremata** | Grapsoidea | Gecarcinidae | *Cardisoma* | *Cardisoma carnifex* | 15597 | MF461623 | [24] |
|  |  |  | Grapsidae | *Pachygrapsus* | *Pachygrapsus crassipes* | 15652 | NC_021754 | [48] |
|  |  |  | Grapsidae | *Pachygrapsus* | *Pachygrapsus marmoratus* | 15406 | MF457403 | [24] |
|  |  |  | Grapsidae | *Grapsus* | *Grapsus tenuicrustatus* | 15858 | NC_029724 | [49] |
|  |  |  | Grapsidae | *Metopograpsus* | *Metopograpsus quadridentatus* | 15517 | MH183127 | [39] |
|  |  |  | Xenograpsidae | *Xenograpsus* | *Xenograpsus testudinatus* | 15798 | EU727203 | [50] |
|  |  |  | Sesarmidae | *Chiromantes* | *Chiromantes neglectum* | 15920 | KX156954 | [51] |
|  |  |  | Sesarmidae | *Sesarmops* | *Sesarmops sinensis* | 15905 | KR336554 | [52] |
|  |  |  | Sesarmidae | *Metopaulias* | *Metopaulias depressus* | 15765 | KX118277 | Unpublished |
|  |  |  | Sesarmidae | *Parasesarma* | *Parasesarma tripectinis* | 15612 | KU343209 | [53] |
|  |  |  | Sesarmidae | *Clistocoeloma* | *Clistocoeloma sinense* | 15706 | KU589292 | [54] |
|  |  |  | Sesarmidae | *Nanosesarma* | *Nanosesarma minutum* | 15637 | MH899177 | This study |
|  |  |  | Varunidae | *Eriocheir* | *Eriocheir japonica hepuensis* | 16335 | FJ455506 | [55] |
|  |  |  | Varunidae | *Eriocheir* | *Eriocheir japonica* | 16352 | NC_011597 | [55] |
|  |  |  | Varunidae | *Eriocheir* | *Eriocheir japonica sinensis* | 16354 | NC_006992 | [17] |
|  |  |  | Varunidae | *Cyclograpsus* | *Cyclograpsus granulosus* | 16300 | LN624373 | [56] |
|  |  |  | Varunidae | *Helice* | *Helice tientsinensis* | 16212 | KR336555 | [57] |
|  |  |  | Varunidae | *Helice* | *Helice latimera* | 14246 | KU589291 | Unpublished |
|  |  |  | Varunidae | *Hemigrapsus* | *Hemigrapsus sanguineus* | 16275 | KX456205 | Unpublished |
|  |  |  | Varunidae | *Helicana* | *Helicana wuana* | 16359 | KX344898 | [58] |
|  |  |  | Varunidae | *Gaetice* | *Gaetice depressus* | 16288 | MH183128 | [39] |
|  |  |  | Varunidae | *Metaplax* | *Metaplax longipes* | 16305 | MH899176 | This study |
|  |  | Ocypodoidea | Macrophthalmidae | *Macrophthalmus* | *Macrophthalmus japonicus* | 16170 | NC_030048 | Unpublished |
|  |  |  | Macrophthalmidae | *Macrophthalmus* | *Macrophthalmus darwinensis* | 16348 | MF457408 | [24] |
|  |  |  | Ocypodidae | *Ocypode* | *Ocypode ceratophthalmus* | 15564 | LN611669 | [59] |
|  |  |  | Ocypodidae | *Ocypode* | *Ocypode cordimanus* | 15604 | NC_029725 | [60] |
|  |  |  | Ocypodidae | *Cranuca* | *Cranuca inversa* | 15677 | MF457405 | [24] |
|  |  |  | Ocypodidae | *Tubuca* | *Tubuca polita* | 15672 | MF457400 | [24] |
|  |  |  | Ocypodidae | *Tubuca* | *Tubuca capricornis* | 15629 | MF457401 | [24] |
|  |  |  | Ocypodidae | *Uca* | *Uca (Gelasimus) borealis* | 15659 | MH183126 | [39] |
|  |  |  | Dotillidae | *Ilyoplax* | *Ilyoplax deschampsi* | 15460 | JF909979 | [18] |
|  |  |  | Dotillidae | Dotilla | *Dotilla wichmani* | 15600 | MH183129 | [39] |
|  |  |  | Mictyridae | *Mictyris* | *Mictyris longicarpus* | 15548 | LN_611670 | [61] |
|  | **Heterotremata** | Leucosioidea | Leucosiidae | *Pyrhila* | *Pyrhila pisum* | 15516 | NC_030047 | [62] |
|  |  | Xanthoidea | Xanthidae | *Leptodius* | *Leptodius sanguineus* | 15480 | KT896744 | [63] |
|  |  | Bythograeidea | Bythograeidae | *Austinograea* | *Austinograea alayseae* | 15620 | JQ035660 | [64] |
|  |  |  | Bythograeidae | *Austinograea* | *Austinograea rodriguezensis* | 15611 | JQ035658 | [64] |
|  |  |  | Bythograeidae | *Gandalfus* | *Gandalfus puia* | 15548 | KR002727 | [65] |
|  |  |  | Bythograeidae | *Gandalfus* | *Gandalfus yunohana* | 15567 | EU647222 | [66] |
|  |  |  | Bythograeidae | *Segonzacia* | *Segonzacia mesatlantica* | 15521 | NC_035300 | [67] |
|  |  | Eriphioidea | Menippidae | *Myomenippe* | *Myomenippe fornasinii* | 15658 | LK391943 | [68] |
|  |  |  | Menippidae | *Pseudocarcinus* | *Pseudocarcinus gigas* | 15515 | AY562127 | [69] |
|  |  |  | Oziidae | *Epixanthus* | *Epixanthus frontalis* | 15993 | MF457404 | [24] |
|  |  | Pilumnoidea | Pilumnidae | *Pilumnus* | *Pilumnus vespertilio* | 16222 | MF457402 | [24] |
|  |  | Calappoidea | Matutidae | *Ashtoret* | *Ashtoret lunaris* | 15807 | LK391941 | [70] |
|  |  | Portunoidea | Geryonidae | *Chaceon* | *Chaceon granulatus* | 16135 | NC_023476 | Unpublished |
|  |  |  | Portunidae | *Scylla* | *Scylla olivacea* | 15723 | FJ827760 | Unpublished |
|  |  |  | Portunidae | *Scylla* | *Scylla paramamosain* | 15824 | JX457150 | [71] |
|  |  |  | Portunidae | *Scylla* | *Scylla serrata* | 15775 | FJ827758 | Unpublished |
|  |  |  | Portunidae | *Scylla* | *Scylla tranquebarica* | 15833 | FJ827759 | Unpublished |
|  |  |  | Portunidae | *Callinectes* | *Callinectes sapidus* | 16263 | AY363392 | [72] |
|  |  |  | Portunidae | *Charybdis* | *Charybdis feriata* | 15660 | KF386147 | [73] |
|  |  |  | Portunidae | *Charybdis* | *Charybdis japonica* | 15738 | FJ460517 | [74] |
|  |  |  | Portunidae | *Charybdis* | *Charybdis natator* | 15664 | NC_036132 | [75] |
|  |  |  | Portunidae | *Portunus* | *Portunus pelagicus* | 16155 | KT382858 | [76] |
|  |  |  | Portunidae | *Portunus* | *Portunus sanguinolentus* | 16024 | KT438509 | [77] |
|  |  |  | Portunidae | *Portunus* | *Portunus trituberculatus* | 16026 | AB093006 | [78] |
|  |  |  | Portunidae | *Thalamita* | *Thalamita crenata* | 15787 | LK391945 | [79] |
|  |  | Majoidea | Oregoniidae | *Chionoecetes* | *Chionoecetes japonicus pacificus* | 15341 | AB_735678 | Unpublished |
|  |  |  | Majidae | *Maja* | *Maja crispata* | 16592 | NC_035424 | [80] |
|  |  |  | Majidae | *Maja* | *Maja squinado* | 16598 | NC_035425 | [80] |
|  |  |  | Mithracidae | *Damithrax* | *Damithrax spinosissimus* | 15817 | KM405516 | [81] |
|  |  | Gecarcinucoidea | Parathelphusidae | *Somanniathelphusa* | *Somanniathelphusa boyangensis* | 17032 | KU042042 | [82] |
|  |  | Potamoidea | Potamidae | *Huananpotamon* | *Huananpotamon lichuanense* | 15380 | KX639824 | [83] |
|  |  |  | Potamidae | *Geothelphusa* | *Geothelphusa dehaani* | 18197 | AB187570 | [84] |
|  |  |  | Potamidae | *Sinopotamon* | *Sinopotamon xiushuiense* | 18460 | KU042041 | [85] |
|  |  |  | Potamidae | *Sinopotamon* | *Sinopotamon yangtsekiense* | 17885 | KY785879 | [22] |
|  |  |  | Potamidae | *Sinopotamon* | *Sinopotamon yaanense* | 17126 | KY785880 | [22] |
| **Outgroups** | |  |  |  |  |  |  |  |
| Anomura | | Galatheoidea | Porcellanidae | *Neopetrolisthes* | *Neopetrolisthes maculatus* | 15324 | NC_020024 | [86] |
|  |  | Galatheoidea | Galatheidae | *Shinkaia* | *Shinkaia crosnieri* | 15182 | NC_011013 | [87] |
|  |  | Paguroidea | Lithodidae | *Lithodes* | *Lithodes nintokuae* | 15731 | NC_024202 | Unpublished |
|  |  | Paguroidea | Lithodidae | *Paralithodes* | *Paralithodes brevipes* | 16303 | NC_021458 | Unpublished |
|  |  | Paguroidea | Paguridae | *Pagurus* | *Pagurus longicarpus* | 15630 | NC_003058 | [88] |
| Gebiidea | | Gebiidea | Upogebiidae | *Austinogebia* | *Austinogebia edulis* | 15761 | NC_019606 | [89] |
|  |  | Gebiidea | Upogebiidae | *Upogebia* | *Upogebia major* | 16119 | JF793665 | [90] |
|  |  | Gebiidea | Thalassinidae | *Thalassina* | *Thalassina kelanang* | 15528 | NC_019608 | [89] |

References

1. Shi GH, Cui ZX, Hui M, Liu Y, Chan TY, Song CW. Unusual sequence features and gene rearrangements of primitive crabs revealed by three complete mitochondrial genomes of Dromiacea. Comparative Biochemistry and Physiology Part D Genomics and Proteomics. 2016; 20:65-73. <https://doi.org/10.1016/j.cbd.2016.07.004>
2. Hui M, Liu Y, Cui Z. First complete mitochondrial genome of primitive crab *Homologenus malayensis* (Decapoda: Brachyura: Podotremata: Homolidae). Mitochondrial DNA. 2016; 27(2):859-860. <https://doi.org/10.3109/19401736.2014.919476>
3. Shi GH, Cui ZX, Hui M, Liu Y, Chan TY, Song CW. The complete mitochondrial genomes of *Umalia orientalis* and *Lyreidus brevifrons*: The phylogenetic position of the family Raninidae within Brachyuran crabs. Marine Genomics. 2015; 21:53-61. <https://doi.org/10.1016/j.margen.2015.02.002>
4. Cheng J, Jiang W, Shi HF, Sha ZL. The complete mitochondrial genome of red frog crab *Ranina ranina* (Crustacea: Decapoda: Brachyura: Raninidae). Mitochondrial DNA. 2016; 27(2):1368-1369. <https://doi.org/10.3109/19401736.2014.947584>
5. Yu YQ, Ma WM, Yang WJ, Yang JS. The complete mitogenome of the lined shore crab *Pachygrapsus crassipes* Randall 1840 (Crustacea: Decapoda: Grapsidae). Mitochondrial DNA. 2014; 25(4):263-264. <https://doi.org/10.3109/19401736.2013.8004971>
6. Sung JM, Lee JH, Kim SK, Zafer Karagozlu M, Kim CB. The complete mitochondrial genome of *Grapsus tenuicrustatus* (Herbst, 1783) (Decapoda, Grapsidae). Mitochondrial DNA Part B. 2016a; 1(1):441-442. <https://doi.org/10.1080/23802359.2016.1180559>
7. Ki JS, Dahms HU, Hwang JS, Lee JS. The complete mitogenome of the hydrothermal vent crab *Xenograpsus testudinatus* (Decapoda, Brachyura) and comparison with brachyuran crabs. Comparative Biochemistry and Physiology Part D. 2009; 4:290-299. https://doi.org/10.1016/j.cbd.2009.07.002
8. Xing YH, Ma XP, Wei YQ, Pan D, Liu WL, Sun HY. The complete mitochondrial genome of the semiterrestrial crab, *Chiromantes neglectum* (Eubrachyura: Grapsoidea: Sesarmidae). Mitochondrial DNA Part B. 2016; 1(1):461-463. <https://doi.org/10.1080/23802359.2016.1186509>
9. Tang BP, Xin ZZ, Liu Y, Zhang DZ, Wang ZF, Zhang HB, et al. The complete mitochondrial genome of *Sesarmops sinensis* reveals gene rearrangements and phylogenetic relationships in Brachyura. Plos One. 2017a; 12(6):e0179800. https://doi.org/10.1371/journal.pone.0179800
10. Park YJ, Park CE, Lee SH, Ko HS, Ullah I, Hwang UW, et al. The complete mitochondrial genome sequence of the intertidal crab *Parasesarma Tripectinis* (Arthropoda, Decapoda, Sesarmidae). Mitochondrial DNA Part B. 2018; 3(1):193-194. <https://doi.org/10.1080/23802359.2018.1437804>
11. Xin ZZ, Liu Y, Zhang DZ, Chai XY, Wang ZF, Zhang HB, et al. Complete mitochondrial genome of *Clistocoeloma sinense* (Brachyura: Grapsoidea): Gene rearrangements and higher-level phylogeny of the Brachyura. Scientific Reports. 2017a; 7(1):4128. <https://doi.org/10.1038/s41598-017-004489-9>
12. Wang J, Huang L, Cheng Q, Lu G, Wang C. Complete mitochondrial genomes of three mitten crabs, *Eriocheir sinensis*, *E. hepuensis*, and *E. japonica*. Mitochondrial DNA Part A. 2016a; 27(2):1175-1176. <https://doi.org/10.3109/19401736.2014.936425>
13. Tan MH, Gan HM, Lee YP, Austin CM. The complete mitogenome of purple mottled shore crab *Cyclograpsus granulosus* H. Milne-Edwards, 1853 (Crustacea: Decapoda: Grapsoidea). Mitochondrial DNA Part A. 2016a; 27(6):3981-3982. <https://doi.org/10.3109/19401736.2014.989514>
14. Xin ZZ, Liu Y, Zhang DZ, Wang ZF, Zhang HB, Tang BP, et al. Mitochondrial genome of *Helice tientsinensis* (brachyura: grapsoidea: varunidae): Gene rearrangements and higher-level phylogeny of the brachyura. Gene. 2017b; 627:307-314. <https://doi.org/10.1016/j.gene.2017.06.036>
15. Tang BP, Liu Y, Xin ZZ, Zhang DZ, Wang ZF, Zhu XY, et al. Characterisation of the complete mitochondrial genome of *Helice wuana* (Grapsoidea: Varunidae) and comparison with other Brachyuran crabs. Genomics (in press). 2017b. <https://doi.org/10.1016/j.ygeno.2017.10.001>
16. Tan MH, Gan HM, Lee YP, Austin CM. The complete mitogenome of the ghost crab *Ocypode ceratophthalmus* (Pallas, 1772) (Crustacea: Decapoda: Ocypodidae). Mitochondrial DNA Part A. 2016b; 27(3):2123-2124.

https://doi.org/10.3109/19401736.2014.982587

1. Sung JM, Lee JH, Kim SG, Zafer Karagozlu M, Kim CB. Analysis of complete mitochondrial genome of *Ocypode cordimanus* (Latreille, 1818) (Decapoda, Ocypodidae). Mitochondrial DNA Part B. 2016b; 1(1):363-364. https://doi.org/10.1080/23802359.2016.1168718
2. Tan MH, Gan HM, Lee YP, Austin CM. The complete mitogenome of the soldier crab *Mictyris longicarpus* (Latreille, 1806) (Crustacea: Decapoda: Mictyridae). Mitochondrial DNA Part A. 2016c; 27(3):2121–2122. <https://doi.org/10.3109/19401736.2014.982585>
3. Park YJ, Chang EP, Jung BK, Ibal JC, Jung YG, Hong SJ, et al. The first complete mitochondrial genome sequence of the leucosiid crab *Pyrhila pisum* (Arthropoda, Decapoda, Leucosiidae). Mitochondrial DNA Part B. 2017; 2(2):885-886. <https://doi.org/10.1080/23802359.2017.1407717>
4. Sung JM, Lee JH, Kim SG, Zafer Karagozlu M, Kim CB. Complete mitochondrial genome of *Leptodius sanguineus* (Decapoda, Xanthidae). Mitochondrial DNA Part B. 2016c;1(1): 500-501. <https://doi.org/10.1080/23802359.2016.1192505>
5. Yang JS, Lu B, Chen DF, Yu YQ, Yang F, Nagasawa H, et al. When did decapods invade hydrothermal vents? Clues from the Western pacific and Indian oceans. Molecular Biology and Evolution. 2013; 30:305-309. https://doi.org/10.1093/molbev/mss224
6. Kim SJ, Moon JW, Ju SJ. Complete mitochondrial genome of the blind vent crab *Gandalfus puia* (Crustacea: Bythograeidae) from the Tonga Arc. Mitochondrial DNA Part A. 2016; 27(4):2719-2720. <https://doi.org/10.3109/19401736.2015.1046162>
7. Yang JS, Nagasawa H, Fujiwara Y, Tsuchida S, Yang WJ. The complete mitogenome of the hydrothermal vent crab *Gandalfus yunohana* (Crustacea: Decapoda: Brachyura): a link between the Bythograeoidea and Xanthoidea. Zoologica Scripta. 2010; 39:621-630.

https://doi.org/10.1111/j.1463-6409.2010.00442.x

1. Mandon P, Aznar-Cormano L, Hourdez S, Samadi S. Assembly of the mitochondrial genome of the hydrothermal vent crab *Segonzacia mesatlantica* and detection of potential nuclear pseudogenes. Mitochondrial DNA Part B. 2017; 2(1):291-293. <https://doi.org/10.1080/23802359.2017.1318674>
2. Tan MH, Gan HM, Lee YP, Austin CM. The complete mitogenome of the stone crab *Myomenippefornasinii* (Bianconi, 1851) (Crustacea: Decapoda: Menippidae). Mitochondrial DNA Part A. 2016d; 27(2):1374-1375. <https://doi.org/10.3109/19401736.2014.947587>
3. Miller AD, Murphy NP, Burridge CP, Austin CM. Complete mitochondrial DNA sequences of the decapod crustaceans *Pseudocarcinus gigas* (Menippidae) and *Macrobrachium rosenbergii* (Palaemonidae). Marine Biotechnology. 2005; 7:339-349. <https://doi.org/10.1007/s10126-004-4077-8>
4. Tan MH, Gan HM, Lee YP, Austin CM. The complete mitogenome of the moon crab *Ashtoret lunaris* (Forskal, 1775), (Crustacea; Decapoda; Matutidae). Mitochondrial DNA Part A. 2016e; 27(2): 1313-1314. <https://doi.org/10.3109/19401736.2014.945572>
5. Ma HY, Ma CY, Li XC, Xu Z, Feng N, Ma LB. The complete mitochondrial genome sequence and gene organization of the mud crab (*Scylla paramamosain*) with phylogenetic consideration. Gene. 2013; 519:120-127. <https://doi.org/10.1016/j.gene.2013.01.028>
6. Place AR, Feng X, Steven CR, Fourcade HM, Boore JL. Genetic markers in blue crabs (*Callinectes sapidus*) II: complete mitochondrial genome sequence and characterization of genetic variation. Journal of Experimental Marine Biology and Ecology. 2005; 319:15–27. <https://doi.org/10.1016/j.jembe.2004.03.024>
7. Ma HY, Ma CY, Li CH, Lu JX, Zou X, Gong YY, et al. First mitochondrial genome for the red crab (*Charybdis feriata*) with implication of phylogenomics and population genetics. Scientific Reports. 2015; 5:11524. <https://doi.org/10.1038/srep11524>
8. Liu Y, Cui ZX. Complete mitochondrial genome of the Asian paddle crab *Charybdis japonica* (Crustacea: Decapoda: Portunidae): gene rearrangement of the marine brachyurans and phylogenetic considerations of the decapods. Molecular Biology Reports. 2010; 37(5):2559-2569. <https://doi.org/10.1007/s11033-009-9773-2>
9. Yang XL, Ma HY, Waiho K, Fazhan H, Wang SQ, Wu QY, et al. The complete mitochondrial genome of the swimming crab *Charybdis natator* (Herbst) (Decapoda: Brachyura: Portunidae) and its phylogeny. Mitochondrial DNA Part B. 2017; 2(2):530-531.
10. Ma CY, Ma HY, Ren GJ, Wang W, Chen W, Lu JX, et al. Characterization of the complete mitochondrial genome of *Portunus pelagicus* with implications for phylogenomics. Genetics and Molecular Research. 2016; 15 (3):gmr.15038719. <https://doi.org/10.4238/gmr.15038719>
11. Ma HY, Ma CY, Zhu JJ, Ren GJ, Wang W, Chen W, et al. Characterization of the complete mitochondrial genome and phylogenetic relationships of the three-spot swimming crab (*Portunus sanguinolentus*). Genetics and Molecular Research. 2016; 15 (3):gmr.15038580. <https://doi.org/10.4238/gmr.15038580>
12. Yamauchi MM, Miya MU, Nishida M. Complete mitochondrial DNA sequence of the swimming crab, *Portunus trituberculatus* (Crustacea: Decapoda: Brachyura). Gene. 2003; 311:129-135. <https://doi.org/10.1016/S0378-1119(03)00582-1>
13. Tan MH, Gan HM, Lee YP, Austin CM. The complete mitogenome of the swimming crab *Thalamita crenata* (Rüppell, 1830) (Crustacea; Decapoda; Portunidae). Mitochondrial DNA Part A. 2016f; 27(2):1275-1276. https://doi.org/10.3109/19401736.2014.945553
14. Basso A, Babbucci M, Pauletto M, Riginella E, Patarnello T, Negrisolo E. The highly rearranged mitochondrial genomes of the crabs *Maja crispate* and *Maja squinado* (Majidae) and gene order evolution in Brachyura. Scientific Reports. 2017; 7:4096. <https://doi.org/10.1038/s41598-017-04168-9>
15. Márquez EJ, Hurtado-Alarcón JC, Isaza JP, Alzate JF, Campos NH. Mitochondrial genome of the Caribbean king crab *Damithrax spinosissimus* (Lamarck, 1818) (Decapoda: Majidae). Mitochondrial DNA Part A. 2016; 27(3):1724-1725. <https://doi.org/10.3109/19401736.2014.961140>
16. Jia XN, Xu SX, Bai J, Wang YF, Nie ZH, Zhu CC, et al. The complete mitochondrial genome of *Somanniathelphusa boyangensis* and phylogenetic analysis of Genus *Somanniathelphusa* (Crustacea: Decapoda: Parathelphusidae). Plos One. 2018; 13(2):e0192601. <https://doi.org/10.1371/journal.pone.0192601>
17. Bai J, Xu SX, Nie ZH, Wang YF, Zhu CC, Wang Y, et al. The complete mitochondrial genome of *Huananpotamon lichuanense* (Decapoda: Brachyura) with phylogenetic implications for freshwater crabs. Gene. 2018; 646:217-226. <https://doi.org/10.1016/j.gene.2018.01.015>
18. Segawa RD, Aotsuka T. The mitochondrial genome of the Japanese freshwater crab, *Geothelphusa dehaani* (Crustacea: Brachyura): evidence for its evolution via gene duplication. Gene. 2005; 355:28-39. <https://doi.org/10.1016/j.gene.2005.05.020>
19. Wang Y, Zeng QW, Zou JX, Zhu CC, Bai J, Shih TH, et al. The complete mitochondrial genome of freshwater crab *Sinopotamon xiushuiense* (Decapoda: Brachyura: Potamoidea). Mitochondrial DNA Part B. 2016b; 1(1):750-752. <https://doi.org/10.1080/23802359.2016.1209094>
20. Shen H, Braband A, Scholtz G. Mitogenomic analysis of decapod crustacean phylogeny corroborates traditional views on their relationships. Molecular Phylogenetics and Evolution. 2013; 66(3):776-789. <https://doi.org/10.1016/j.ympev.2012.11.002>
21. Yang JS, Yang WJ. The complete mitochondrial genome sequence of the hydrothermal vent galatheid crab *Shinkaia crosnieri* (Crustacea: Decapoda: Anomura): A novel arrangement and incomplete tRNA suite. BMC Genomics. 2008; 9:257. <https://doi.org/10.1186/1471-2164-9-257>
22. Hickerson MJ, Cunningham CW. Dramatic Mitochondrial Gene Rearrangements in the Hermit Crab *Pagurus longicarpus* (Crustacea, Anomura). Molecular Biology and Evolution. 2000; 17(4):639-644.

https://doi.org/10.1093/oxfordjournals.molbev.a026342

1. Lin FJ, Liu Y, Sha Z, Tsang LM, Chu KH, Chan TY, et al. Evolution and phylogeny of the mud shrimps(Crustacea: Decapoda) revealed from complete mitochondrial genomes. BMC Genomics. 2012; 13(1):631. <https://doi.org/10.1186/1471-2164-13-631>
2. Kim S, Kim T, Choi HG, Park JK, Ahn DH, Min GS. The complete mitochondrial genome of the Japanese mud shrimp *Upogebia major* (Crustacea, Decapoda). Mitochondrial DNA. 2011; 22(4): 94-96. https://doi.org/10.3109/19401736.2011.624609
