# Supplementary material for: Phylomitogenomics reconfirm the phylogenetic position of the genus Metaplax inferred from the two grapsid crabs (Decapoda: Brachyura: Grapsoidea)
Source: PLoS One. 2019 Jan 25;14(1):e0210763. doi: 10.1371/journal.pone.0210763 (PMC6347246; doi:10.1371/journal.pone.0210763)
Supplement: S2 Table — (DOCX) [file pone.0210763.s002.docx]

**Table S2. The lengths and A+T contents of the mitogenomes of 33 taxa from the Grapsoidea and Ocypodoidea.**

| Species | Size (bp) | A % | G % | T % | C % | A+T % |
| --- | --- | --- | --- | --- | --- | --- |
| *Pachygrapsus crassipes* | 15,652 | 30.50 | 12.70 | 35.80 | 21.00 | 66.30 |
| *Grapsus tenuicrustatus* | 15,858 | 31.90 | 12.10 | 33.10 | 22.80 | 65.00 |
| *Metopograpsus quadridentatus* | 15,517 | 34.30 | 10.20 | 36.00 | 19.50 | 70.30 |
| *Xenograpsus testudinatus* | 15,798 | 36.70 | 9.30 | 37.20 | 16.80 | 73.90 |
| *Chiromantes neglectum* | 15,920 | 37.40 | 9.50 | 38.20 | 14.90 | 75.60 |
| *Sesarmops sinensis* | 15,905 | 37.40 | 9.40 | 38.30 | 14.90 | 75.70 |
| *Metopaulias depressus* | 15,765 | 37.90 | 8.70 | 39.40 | 14.00 | 77.30 |
| *Parasesarma tripectinis* | 15,612 | 36.20 | 10.10 | 38.00 | 15.70 | 74.20 |
| *Clistocoeloma sinense* | 15,706 | 37.10 | 9.40 | 38.60 | 14.90 | 75.70 |
| *Nanosesarma minutum* | 15,637 | 38.00 | 8.90 | 39.70 | 13.40 | 77.70 |
| *Eriocheir japonica hepuensis* | 16,335 | 35.10 | 10.80 | 36.40 | 17.70 | 71.50 |
| *Eriocheir japonica* | 16,352 | 35.20 | 10.70 | 36.50 | 17.70 | 71.70 |
| *Eriocheir japonica sinensis* | 16,378 | 35.20 | 10.80 | 36.40 | 17.60 | 71.60 |
| *Gaetice depressus* | 16,288 | 35.40 | 10.50 | 37.60 | 16.50 | 73.00 |
| *Cyclograpsus granulosus* | 16,300 | 33.20 | 11.20 | 36.10 | 19.50 | 69.30 |
| *Helice tientsinensis* | 16,212 | 33.90 | 11.00 | 35.10 | 19.90 | 69.00 |
| *Helice latimera* | 16,246 | 34.00 | 11.00 | 35.10 | 19.90 | 69.10 |
| *Hemigrapsus sanguineus* | 16,275 | 34.30 | 11.20 | 35.50 | 19.10 | 69.80 |
| *Metaplax longipes* | 16,305 | 37.60 | 10.60 | 33.80 | 17.90 | 71.40 |
| *Helicana wuana* | 16,359 | 33.00 | 11.50 | 35.50 | 20.00 | 68.50 |
| *Macrophthalmus japonicus* | 16,170 | 33.60 | 10.90 | 32.80 | 22.70 | 66.40 |
| *Ocypode ceratophthalmus* | 15,564 | 33.70 | 11.10 | 35.80 | 19.40 | 69.50 |
| *Ocypode cordimanus* | 15,604 | 31.80 | 11.90 | 34.50 | 21.80 | 66.30 |
| *Uca (Gelasimus) borealis* | 15,659 | 35.00 | 11.50 | 34.40 | 19.10 | 69.40 |
| *Ilyoplax deschampsi* | 15,460 | 34.10 | 10.70 | 35.50 | 19.70 | 69.60 |
| *Dotilla wichmani* | 15,600 | 33.80 | 10.80 | 34.70 | 20.70 | 68.50 |
| *Mictyris longicarpus* | 15,548 | 32.40 | 11.80 | 36.60 | 19.20 | 69.00 |
| *Cardisoma carnifex* | 15,597 | 35.40 | 10.10 | 33.40 | 21.00 | 68.80 |
| *Pachygrapsus marmoratus* | 15,406 | 31.40 | 12.10 | 37.00 | 19.50 | 68.40 |
| *Macrophthalmus darwinensis* | 16,348 | 34.30 | 11.10 | 34.00 | 20.70 | 68.30 |
| *Cranuca inversa* | 15,677 | 35.80 | 10.90 | 35.20 | 18.10 | 71.00 |
| *Tubuca polita* | 15,672 | 36.60 | 10.90 | 35.00 | 17.50 | 71.60 |
| *Tubuca capricornis* | 15,629 | 35.50 | 11.40 | 35.00 | 18.10 | 70.50 |
